# Efficacy of a Digital Health Tool on Contraceptive Ideation and Use in Nigeria: Results of a Cluster-Randomized Control Trial

**DOI:** 10.9745/GHSP-D-19-00066

**Published:** 2019-06-24

**Authors:** Stella Babalola, Caitlin Loehr, Olamide Oyenubi, Akinsewa Akiode, Allison Mobley

**Affiliations:** a Johns Hopkins Center for Communication Programs, Baltimore, MD, USA.; b IntraHealth International, Washington, DC, USA.; c Nigeria Urban Reproductive Health Initiative, Abuja, Nigeria.; d Consultant, Baltimore, MD, USA.

## Abstract

**Background:**

Contraceptive prevalence in Nigeria remains among the lowest in the world, which substantially contributes to the country's high maternal and child mortality. Mobile phone technology penetration has increased considerably in Nigeria, opening opportunities for programs to use this medium for reaching their intended audience with health-protective information.

**Methods:**

In 2017, the Health Communication Capacity Collaborative conducted a cluster-randomized control trial in Kaduna City to assess the efficacy of the digital health tool Smart Client on ideational and behavioral variables related to family planning. Twelve wards in the city were randomly assigned to intervention (6 wards) and control (6 wards) arms of the study. A total of 565 women aged 18–35 years were randomly selected from study wards and consented to participate in the study. At recruitment, the women completed a baseline survey. The women in the intervention group were registered to receive 1 welcome call, 13 program calls, and 3 quiz calls on their mobile phones. Each of the program calls had several segments, including introduction, drama episode, and friend-to-friend chat. The last quiz call included evaluation questions. Women in the control arm received no intervention. The efficacy of the intervention was assessed using both per-protocol and intent-to-treat differences-in-differences techniques.

**Results:**

The intervention and control arms were equivalent in terms of key sociodemographic characteristics, with the exception of religion. Attrition was a major challenge in the study. On average, participants receiving the intervention listened to 7.2 drama episodes but only 2.6 personal stories and 1.1 sample dialogues. The results of both per-protocol and intent-to-treat analyses show that the intervention was efficacious in improving relevant ideational and behavioral outcomes. For example, the intent-to-treat results show that the intervention increased women's perceived level of confidence to discuss family planning with a provider by 27.7 percentage points and modern contraceptive prevalence by 14.8 percentage points.

**Conclusion:**

This efficacy assessment showed that using an interactive voice response-based digital tool that includes drama is a viable option for promoting positive ideation about family planning and increasing contraceptive use in Nigeria. Significant lessons learned from this efficacy trial include informing participants at the time of recruitment of what the opening segment of the calls will sound like to avoid the calls being mistaken for telemarketing calls and intensive testing prior to scale-up to avoid potential attrition due to technical issues.

## Abstract (precis)

A mobile digital health tool piloted in Kaduna City, Nigeria, was efficacious in promoting positive contraceptive attitudes and encouraging women to adopt a modern contraceptive method, thus showing potential for reducing unmet need in Nigeria.

## BACKGROUND

Despite decades of government and donor investments in family planning service provision and demand generation, the contraceptive prevalence rate in Nigeria remains one of the lowest in the world. Findings from the 2016–2017 Multiple Indicator Cluster Survey revealed that only 13.4% of in-union women of reproductive age were using any contraceptive method, while only 10.8% reported using a modern method.^1^ Compared with data from the 1999, 2003, and 2013 Demographic and Health Surveys, these latest survey results indicate that contraceptive prevalence in Nigeria has not increased meaningfully over the last 20 years.^2^^,^^3^ Huge differences among the states and regions have also persisted. For example, in 2016, modern contraceptive prevalence varied between 0.2% in Yobe state in the northeast and 30.0% in Oyo state in the southwest.^1^ Low contraceptive use is a key contributor to the high levels of fertility, maternal mortality, and infant mortality observed in Nigeria.^4^^,^^5^ Indeed, contraception has long been recognized as a crucial intervention for improving maternal and child health outcomes and economic opportunities for women.^6^^,^^7^ Use of contraceptive methods has also been shown to positively influence gender-related dynamics and economic empowerment for women.^8^^–^^10^

Lack of access is a key barrier to contraceptive use among Nigerian women. Studies have identified several other demand- and supply-side factors that affect contraceptive use in Nigeria and elsewhere in Africa. Literature abounds on the role of supply-side factors including service availability, distance to services, cost of services, and provider bias, interpersonal skills, and technical competence.^11^^–^^16^ On the demand side, sociodemographic characteristics of the women (e.g., age, parity education, religion), household and conjugal variables (e.g., partner's characteristics, household wealth, type of marriage, woman's autonomy), and community norms have also been shown to affect contraceptive use.^17^^–^^24^ The extant literature contains evidence of the important role of ideational (psychosocial) variables in the decision to use contraceptives. The ideational variables that have been found to be associated with contraceptive use include contraceptive awareness, attitudes, spousal communication, perceived self-efficacy to take actions related to contraceptive use, fear of side effects, misconceptions about contraceptives, perceived social support for contraception, and family size ideals.^19^^,^^25^^–^^28^

For men and women, adopting a contraceptive method is often the result of a process that involves discussions and decision making about whether to practice family planning, what method to use, where to obtain it, and whether to continue using it. Throughout this process, a person's fertility desires, perceptions about contraceptives, efficacy beliefs, and perceived social support for family planning may affect the decisions taken. The person may seek out information on family planning, talk with their partner, and discuss experiences with family and friends. At some point in this process, the person is likely to visit with a family planning provider.

Communication is a core skill running throughout this decision-making process—communicating with one's partner, family and friends, and a health care provider. In most parts of Nigeria, however, communicating about sex, fertility desires, and use of family planning methods is not culturally appropriate.^29^^,^^30^ Furthermore, women and men often lack the skills they need to communicate effectively about such personal and sensitive subjects. Family planning demand generation programs are often designed to address the ideational, normative, and health systems-related factors that hinder women and men from seeking family planning services and adopting contraception. The programs rarely focus on preparing clients to be active and engaged communicators during their interactions with the family planning service provider. In many countries and settings, efforts made to improve providers' communication skills and provide client-centered counseling have led to some improvement in client engagement, but the client is still often dependent on the provider to lead this process.^31^^,^^32^ As a result, female clients are often passive participants in family planning counseling, resulting in discussion and decision making being led by the provider.

The Health Communication Capacity Collaborative (HC3) was a 5-year project implemented between 2012 and 2017 by the Johns Hopkins Center for Communication Programs with funding from the United States Agency for International Development (USAID). One of the focal areas of HC3 was strengthening the capacity of its partners in developing countries to implement effective social and behavior change communication programs to improve contraceptive uptake. In this regard, the HC3 family planning team developed a digital tool, *Smart Client* or *Beta Life* in Nigeria, for implementing partners to use to increase the number of family planning clients who are informed, empowered, and confident. These “smart clients” are expected to be able to engage with providers and talk about their family planning needs. The *Smart Client* tool is inspired by earlier works on family planning clients' coaching.^33^^–^^36^ The global proliferation of mobile technologies has led to their successful use for enhancing women's knowledge about their health and increasing demand for various health services in many countries, including Bangladesh, Cambodia, Ghana, Kenya, Pakistan, South Africa, and Tanzania. Indeed, mobile phone-based interventions have been documented to promote behaviors related to child immunization,^37^ prevention of mother-to-child transmission of HIV,^38^ newborn health,^39^ pregnancy and infant care,^40^^–^^45^ and family planning.^46^^,^^47^ With digital health interventions, it is possible to reach a large segment of the intended audience with targeted health information. Moreover, digital tools can offer a more personal and private experience, which is particularly important in accessing information on a sensitive subject such as family planning. A woman may be hesitant to listen to a radio or television program because others might hear it as well, or she may not attend a community event for similar reasons. However, mobile phone-based approaches can offer users a private space to access information. The advantages of digital health are particularly relevant in low-resource settings and for people with limited access to the traditional sources of health information (radio, television, community events), including women and other marginalized groups. Mobile phone penetration, both in terms of active subscriptions and unique subscribers, has increased conspicuously in Nigeria in recent years: from 23% of the population being unique subscribers in 2010 to 49% in 2017.^48^^,^^49^ This high level of penetration makes the use of this technology potentially effective for disseminating health information across various audience groups in the country.

Digital tools can offer a more private experience, which is important in accessing information on sensitive subjects such as family planning.

The HC3 family planning team leveraged the power of basic mobile technology to develop a digital health tool to prepare women to become smart clients and encourage them to talk with their provider and partner about contraceptive methods. Here we report on the findings of the pilot test of the tool in Kaduna City, Nigeria. The analyses assess the effects of exposure to the digital tool on contraceptive ideation and use among women of reproductive age.

## THE INTERVENTION

The *Smart Client* digital health tool was designed to inform, empower, and promote smart clients by reaching them directly through mobile phones. The tool is based upon Social Learning Theory, which posits that people learn from each other through observation, imitation, and modeling. The *Smart Client* tool therefore uses fictional role models, who demonstrate the desired behaviors and behavior change process in a drama format, as well as personal stories and examples of smart client dialogues. This approach allows the intended audience to observe an action, understand its consequences, and become motivated to repeat and adopt it. While drama is a common approach used in behavior change communication, it is usually delivered via television, radio, or community theater. This digital health tool explored how drama could be adapted to basic mobile phones via interactive voice response (IVR), using shorter and simpler storylines in a series of episodes while maintaining the fictional serial drama style. IVR was chosen as the delivery channel because it is accessible to audiences regardless of the type of mobile phone they have (e.g., smartphone or basic phone) and irrespective of their level of literacy. Given the lack of evidence for delivering entertainment-education content via IVR with the aim of promoting behavior change, the HC3 project tested and evaluated this approach.

The *Smart Client* digital tool is based upon Social Learning Theory, which posits that people learn from each other.

The *Smart Client* digital health tool was designed to be delivered via mobile phone and included 17 prerecorded calls: 1 welcome call, 13 regular program calls, and 3 quiz calls interspersed. The order of the calls is outlined in the Box. Each regular program call includes 5 segments:

1. Brief welcome in which the host characters provide an introduction to the short drama included in the call
2. A short drama that follows a couple, Laila and Musa, and some of their friends as they face challenges and make decisions about contraceptive use
3. A “friend-to-friend chat” in which the hosts reinforce the key messages included in the drama segment and ask the user a quiz question
4. An optional personal story segment that requires the listener to use the keyboard to indicate whether they are interested in listening. Personal stories focus on diverse experiences related to family planning that correspond to the key message of the short drama episode.
5. Sample dialogue is another optional segment that features a friendly provider and a client, modeling what to expect during a family planning clinic visit and how to discuss needs, preferences, and concerns.

BOX **Description of the *Smart Client* Program**

**Call 1: Welcome call.** The users listen to an introduction about the tool explaining how it works and what to expect from the content. They answer 3 questions regarding age, frequency, and time to receive calls using their numeric keypad. The pre-intervention questions were posed at the end of this call and study participants used their numeric keypad to respond.

**Calls 2–7: Regular calls.** These are part of the 13 regular calls. Users listen to the first 3 segments and then can choose to listen to any of the optional segments:

1. **Brief welcome and introduction** to the story by friendly host characters, a female and male.
2. **Short episode of the serial drama,** which follows a cast of characters including a couple, Laila and Musa, along with their family and friends, who all face different situations and challenges related to using family planning methods.
3. **“Friend-to-friend” chats,** in which the host “friends” deliver follow-up messages and tips related to the core message and the drama and ask the user a quiz question. Some messages in this segment are tailored for male and female users, based on their user preferences set on enrollment, or tailored to the user response to the question.
4. **Personal story.** This segment is optional, requiring users to “press 1” to hear the content. Personal stories, told by females and males, express diverse experiences with family planning that correspond to the key message of the episode.
5. **Sample dialogue.** This segment is also optional, requiring users to “press 2” to hear the content. Sample dialogues feature a friendly provider and a client or a couple, modeling what to expect during a visit to a family planning clinic and how to discuss needs, preferences, and concerns.
6. **Wrap up and quiz question.** To conclude the call, the hosts repeat key messages, ask users 1 to 2 question(s) to evaluate understanding of key messages, and sign off with a reminder to listen to the next call in the series.

**Call 8: Short quiz call.** Participants listen to the host ask 4 questions and answer these questions using their numeric keypad.

**Calls 9–11: Regular calls.** These are part of the 13 regular calls. Users listen to the first 3 segments and then can choose to listen to any of the optional segments as described above.

**Call 12: Short quiz call.** Users listen to the host ask up to 5 questions and answer using their numeric keypad.

**Calls 13–16: Regular calls.** These are part of the 13 regular calls. Users listen to first 3 segments and then can choose to listen to any of the optional segments as described above.

**Call 17: Short quiz call.** Users listen to the host ask 6 questions and answer using their numeric keypad. The post-intervention questions were asked at the end of this call and study participants used their numeric keypad to respond.

During the 3 short quiz calls, participants were asked a few brief questions to reinforce key messages, evaluate user understanding of content, and encourage user engagement. In addition, users received a short message service (SMS) reminder about the key message from each call.

The tool and its content were pretested in a focus group discussion setting among a group of women representative of the intended audience in Kaduna. Pretest participants shared feedback on the content—whether it was realistic, acceptable, and relevant—as well as their overall perceptions about the tool. The feedback served as the basis for finalizing the tool and its content prior to production and rollout. For the efficacy testing reported in this manuscript, the tool was given the name *Beta Life*, the content of the digital health tool was recorded in Hausa, and all SMS messages were written in Hausa.

The IVR platform was programmed so that users were preregistered and calls would be pushed to them on a schedule (every day, every other day, or twice per week) and time of day (morning, afternoon, or evening) of their choice. If the call came at an inconvenient time or if the user wanted to relisten to a call, they had the option of “flashing” or initiating a dropped call to the platform phone number and they would be immediately called back and could listen to the call that they missed or most recently listened to. For the purposes of the pilot test, users were not able to listen to calls out of sequence.

## METHODS

### Study Design and Data

A cluster-randomized control trial was used for this study. Clusters (wards of residence) were randomly assigned to one of two intervention conditions: receive the digital intervention or receive nothing. The required sample size was determined based on the proportion of women who had discussed contraceptive use with their spouse in the last 12 months. Since the value of this indicator was unknown in the study population, we assumed it to be 50% in our calculations since this level provided maximum variability. We also assumed that this indicator would increase by 15 percentage points among the women in the intervention group, and the required sample size was therefore 240 women for each arm. Estimating a loss to follow-up rate of 20%, we deemed it necessary to recruit 300 women into each arm. This number would provide a 90% power to detect a difference of 15 percentage points between the intervention and the control groups in the proportion of women who had discussed family planning with their husband or partner.

Clusters (wards of residence) were randomly assigned to one of two intervention conditions: receive the digital intervention or receive nothing.

To recruit women into the study, we randomly selected 6 wards from each of the 2 local government areas (LGAs) in Kaduna metropolis—Kaduna North and Kaduna South. The study wards have comparable access to family planning services. Three wards from each local government area were randomly assigned to the intervention group and 3 to the control group. Trained female field agents, fluent in Hausa, went door to door in sample wards to identify eligible women, explain the purpose and method of the study, obtain informed consent, and recruit participants.

Consenting participants in the intervention group were registered to receive the *Smart Client* calls. The pre-intervention survey for the intervention group was administered as an automated survey at the end of the first call. The post-intervention survey for this group was also an automated survey that directly followed the last call, between 3 and 11 weeks after they started the intervention depending on the frequency of the calls. The control arm did not receive the *Smart Client* intervention but received 2 calls on their mobile phone: one at the beginning of the study with the automated pre-intervention survey and the other 6 weeks later with the automated post-intervention survey.

At the time of recruitment, after informed consent was obtained in person, each participant completed a pre-study questionnaire to provide information on her age, number of children, religion, marital status, LGA and ward of residence, address, preferred nickname to be used during the study, primary and secondary cell phone numbers, and whether she shares a phone with anyone. Data from the pre-intervention and post-intervention survey calls, as well as user analytics collected by the IVR platform, were combined with pre-study data to conduct our analyses.

### Setting and Participants

The study took place in North and South Kaduna LGAs of Kaduna State, Nigeria, from March 7, 2017, to June 5, 2017. The 2 LGAs are urban and make up the Kaduna metropolis. Residents included a mixture of Muslims and Christians, although the residents of Kaduna North are predominantly Muslim, while Kaduna South is predominantly Christian. Kaduna metropolis had an estimated total of 1.3 million inhabitants in 2017 and is a melting pot for various Nigerian ethnic groups. While the predominant ethnic group in the city is Hausa, the metropolis also includes large proportions of Yoruba, Igbo, Fulani, Gbaju, and other Nigerian ethnic groups. Secondary analysis performed by the lead author of survey data collected by Measurement Learning & Evaluation, in 2015^50^ revealed that the majority (78.6%) of the women in the city had postprimary education while one-fifth had tertiary education. In the same survey, 21.0% of women of reproductive age reported using a modern contraceptive method, while 6.5% reported using a traditional method.

The intended audience for the digital tool is women of reproductive age. As such, women eligible for recruitment into the study were those with the following characteristics: aged between 18 and 35 years and not currently using a nonbarrier contraceptive method (e.g., pill, intrauterine device, implant, emergency contraceptives, tubal ligation, vasectomy, Lactational Amenorrhea Method), owned a mobile phone or had access to one, resident in Kaduna City, and fluent in Hausa.

The intended audience for the digital tool is women of reproductive age.

### Variables

The ideational and behavioral outcomes assessed in this manuscript include the following:

1. Considerations for desired family size—defined as having ever given thought to the number of children desired
2. Perceive self-efficacy for communicating with a family planning provider—defined as reporting a high level of confidence in one's ability to discuss one's concerns about contraceptives with a provider
3. Spousal communication about family size—defined as discussion of desired family size with one's spouse in last 6 months
4. Spousal communication about contraceptive methods—defined as discussion of contraceptive methods with one's spouse in last 6 months
5. Misinformation rejection—defined as rejection of the misconception that contraceptives can harm the womb
6. Current modern contraceptive use—defined as currently using any modern contraceptive method

### Statistical Analysis

Data from the automated surveys (e.g., pre-intervention and post-intervention) and user analytics (e.g., number of calls received, number of episodes and segments completed) were combined with the demographic information collected at the time of recruitment and analyzed using summary statistics to compare ideational and behavioral outcomes among participants in the intervention or control groups. To assess the short-term effects of the digital health tool, difference-in-differences (DID) analytic method was employed. Note that each relevant outcome is measured in both the intervention and the control groups at 2 points in time: at the beginning and at the end of the study. DID evaluates the significance of the difference in gains over time between the intervention and control groups. More formally, the DID model is as follows:

$$\delta =\left({\text{Y}}_{1\text{p}}-{\text{Y}}_{1\text{c}}\right)-\left({\text{Y}}_{0\text{p}}-{\text{Y}}_{0\text{c}}\right)$$

where δ is the difference-in-difference estimator; Y_1p_ is the relevant outcome at the end of the study for the intervention group; Y_0p_ is the relevant outcome at the beginning of the study for the intervention group; Y_1c_ is the relevant outcome at the end of the study for the control group; and Y_0c_ is the relevant outcome at the beginning of the study for the control group.

To strengthen the claim about the causal effect of the tool on assessed outcomes, the analyses controlled for relevant sociodemographic variables in the estimation of DID. Specifically, the estimation models controlled for the following variables: religion, current age, parity, education, marital status, and number of days elapsed between the pre-study interview and the end of the study interview.

The findings reported in this manuscript were derived from both per-protocol and intention-to-treat DID. In the per-protocol DID, only participants that met eligibility criteria were recruited into the study and only those that completed the post-study assessment were included in the analysis. The choice to do per-protocol analysis was due to the high level of attrition and because of the heterogeneity between the women who participated in the post-study survey and their peers that were lost to follow-up. Nonetheless, in conformity with CONSORT (Consolidated Standards of Reporting Trials) recommendations^51^, we also performed intention-to-treat analysis with all the women recruited into the study and who participated in the baseline survey. In the intention-to-treat analysis, eligible and recruited participants are included in the analysis, irrespective of whether they completed the post-study. The outcome was not measured for women who did not participate in the endline survey. For the intention-to-treat analyses, at post-study, baseline responses to ideational and behavioral questions were attributed to the women lost to follow-up since these responses were the most recent and only outcome information that we had for them. The significance of the intention-to-treat analysis should strengthen the claim about the efficacy of the intervention.

The findings reported in this article were derived from both per-protocol and intention-to-treat DID.

Furthermore, for the intervention group, user analytics were analyzed to track usage patterns (e.g., number of calls, average length of time listened to segments, navigation patterns, number of questions answered in quizzes, number of episodes heard) and gather general feedback on the user experience with the tool.

### Ethical Considerations

The study was approved by the Johns Hopkins Bloomberg School of Public Health Institutional Review Board and by the National Health Research Ethics Committee in Nigeria. Study participants gave informed consent prior to their participation in the study. Every participant was made to understand that participation was entirely voluntary and that they could choose not to participate at any time. At the completion of the study and consistent with what was stated in the consent script, all intervention participants who listened to any part of the final call and control participants who listened to any part of the post-intervention survey received an incentive of a nominal amount of airtime credit equivalent to US$1.50 for their participation in the study.

## RESULTS

### Sociodemographic Characteristics of Study Participants

Table 1 compares the sociodemographic characteristics of the intervention and control groups. Overall, the average age was 26.8 years, and no significant difference existed between the intervention group (26.4 years) and the control group (27.0 years). The intervention and control groups were also equivalent in terms of marital status, education, and parity. In contrast, there were significant differences by religion. Specifically, a larger proportion of the intervention group was Muslims (65.6%) compared with the control group (65.6% vs. 57.2%, respectively; *P*<.05).

**TABLE 1.** Sociodemographic Characteristics of Study Participants Before the Intervention, by Study Group, Kaduna, Nigeria, 2017

Sociodemographic Indicator	Both Groups (N=565)	Intervention Group (n=221)	Control Group (n=344)	*Z* (or *t*)/*P* for Difference Between Groups
Mean age, years	26.8	26.4	27.0	1.380/.17
Currently married, %	55.9	53.4	57.5	0.973/.33
Tertiary education, %	32.0	33.0	31.4	0.407/.68
Muslim, %	60.5	65.6	57.2	1.980/.048
Mean parity	2.36	2.22	2.44	1.109/.27

The intervention and control groups were equivalent in terms of marital status, education, and parity but differed by religion.

### Exposure to the Intervention

User analytics data captured by the platform showed that the duration of listening differed between calls, which can be partially explained by variation in the content of the calls. The average listening times varied from less than 2 minutes to more than 10 minutes. Quiz calls 8 and 12 had the shortest average listening times. The longest average listening time was for the first call (10 minutes), which included the introduction and the pre-intervention survey, and call 17 (almost 11 minutes), which included the last quiz and the post-intervention survey. The average listening duration across all calls with program content was 5 minutes and 26 seconds.

The data further showed that the majority (96%) of the women in the intervention group listened to at least 1 complete episode of the drama—defined as a user listening to 100% of the episode. The episodes most likely to have been heard in their entirety by the study participants were the first 3 episodes, whereas exposure was relatively lower for the last 3 of the 13 drama episodes. The average number of episodes completely heard was 7.2.

Table 2 describes variations in exposure to the various program components by key sociodemographic characteristics of the women in the intervention group. The data showed no differences in exposure to complete drama episodes by level of education or age group. On the other hand, exposure varied significantly by parity, religion, and marital status. On average, Muslim participants completed more drama episodes than Christian participants did (7.83 vs. 6.11, respectively; *P*<.001). Similarly, ever-married women completed more episodes, on average, than their never-married peers (7.69 vs. 6.63, respectively; *P*<.05).

**TABLE 2.** Mean Number of *Smart Client* Drama Episodes Completed, by Sociodemographic Characteristics, Intervention Group, Kaduna, Nigeria, 2017 (n=221)

Sociodemographic Characteristics	Mean No. of Drama Episodes Completed	Mean No. of Personal Stories Completed	Mean No. of Sample Dialogues Completed
**Age group, years**			
18–24	6.89	2.33	1.81
25+	7.45	2.75	2.01
**Education level**			
Secondary or less	7.28	2.60	2.13*
Tertiary	7.17	2.58	1.53
**Marital status**			
Never married	6.63*	2.13*	1.25***
Ever married	7.69	2.93	2.44
**Religion**			
Muslim	7.83***	2.95**	2.40***
Christian	6.11	1.89	1.04
**Parity**			
0–1	6.65*	2.11*	1.25***
≥2	7.65	2.92	2.40
**All**	7.24	2.59	1.15

* *P*<.05;

** *P*<.01;

*** *P*<.001.

Compared with the drama series episodes, exposure to the personal stories and the sample dialogues was lower. The participants in the intervention group listened to 2.59 complete personal stories and 1.15 sample dialogues, on average. Similar to what the data on the drama series episodes showed, exposure to the personal stories and the sample dialogues varied significantly by marital status, religion, parity, and education level (Table 2).

### Efficacy of the Program

The results of the DID analyses are provided in Table 3 and Table 4. The efficacy of the tool was initially assessed among the participants that answered the post-study questions (per-protocol analyses). A broader analysis was also conducted, focusing on the total sample and using intention-to-treat techniques. The results of the DID estimation adjusted for the participants' age, education, religion, parity, and marital status (where appropriate). Notably, the women who participated in the post-study survey had a significantly higher level of exposure to the tool than their peers who did not participate in the post-study survey. For example, the women who participated in the post-study were exposed to a significantly higher mean number of drama series episodes (9.30) than their peers who did not participate in the post-study interview (5.75). In other words, the per-protocol effects reported below are probably indicative of what could be expected in the context of a high level of exposure to the tool by a wider audience.

**TABLE 3.** Change in Selected Ideational and Behavioral Outcomes and Results of Differences-in-Differences (DID), per-Protocol Analyses, Kaduna, Nigeria, 2017^a^

Intervention Condition	Percent Reporting Outcome		DID Results		
	Pre-Study	Post-Study	Estimate in Percentage Points	Std. Error	*P*
**Already thought of the number of children to have**					
Intervention group	33.0	77.5	43.2	.087	<.001
Control group	42.5	43.8			
**Confident discussing family planning with provider**					
Intervention group	35.5	73.6	61.5	.089	<.001
Control group	59.5	36.1			
**Discussed family size with spouse in last 6 months (currently married women only)**					
Intervention group	65.2	98.5	41.2	.118	.001
Control group	74.6	66.7			
**Discussed contraceptive methods with spouse in last 6 months (currently married women only)**					
Intervention group	46.4	75.8	22.7	.119	.06
Control group	43.0	49.7			
**Rejected the myth that contraceptive methods can hurt a woman's womb**					
Intervention group	50.6	78.8	48.4	.089	<.001
Control group	64.1	43.9			
**Using modern contraceptive method**					
Intervention group	28.8	63.6	34.8	.084	<.001
Control group	32.7	32.7			

a Total sample size (per protocol): intervention (n=92); control (n=158). All estimated models controlled for age, education, number of children ever born, and education.

**TABLE 4.** Change in Selected Ideational and Behavioral Outcomes and Results of Differences-in-Differences (DID), Intention-to-Treat Analyses, Kaduna, Nigeria, 2017^a^

Intervention Condition	Percent Reporting Outcome		DID Results		
	Pre-Study	Post-Study	Estimate in Percentage Points	Std. Error	*P*
**Already thought of the number of children to have**					
Intervention group	24.8	43.5	17.8	.052	<.001
Control group	24.5	25.4			
**Confident discussing family planning with provider**					
Intervention group	20.4	36.3	27.7	.054	<.001
Control group	30.0	18.2			
**Discussed family size with spouse in last 6 months (currently married women only)**					
Intervention group	23.6	32.9	15.4	.073	.03
Control group	32.1	26.0			
**Discussed contraceptive methods with spouse in last 6 months (currently married women only)**					
Intervention group	17.3	30.0	9.6	.074	.18
Control group	16.5	19.6			
**Rejected the myth that contraceptive methods can hurt a woman's womb**					
Intervention group	25.2	37.0	22.7	.057	<.001
Control group	30.4	19.5			
**Using modern contraceptive method**					
Intervention group	22.9	37.4	14.8	.050	.003
Control group	20.9	20.6			

a Total sample size (intention to treat): intervention (n=220); control (n=339). All estimated models controlled for age, education, number of children ever born, and education.

Following is a presentation of the results for each outcome:

1. **Thoughts about desired family size.** The per-protocol analysis shows that at pre-study, women in the control group were more likely than their peers in the intervention group to have ever thought of their desired family size (42.5% compared to 33.0%). At the post-study, essentially no change had occurred in the control group. However, proportionally more women in the intervention group reported having ever given thought to the desired family size. The per-protocol DID estimate shows that the intervention led to a significant 43.2 percentage point increase in this indicator (Table 3). Results of the intention-to-treat analysis reveal a lower, albeit significant effect of 17.8 percentage points (Table 4).
  Both the per-protocol and intention-to-treat analyses showed that the intervention led to a significant increase in thinking about desired family size.
2. **Confidence in one's ability to discuss concerns about contraceptive methods with a provider.** According to the results of the per-protocol analysis, between pre-study and post-study, the proportion of participants confident in their ability to discuss concerns about contraceptive methods with a provider increased significantly in the intervention group (from 35.5% to 73.6%), whereas it declined conspicuously in the control group (from 59.5% to 36.1%). The reason for the huge decline among control group members is not clear. Results of the per-protocol DID estimation reveal a 61.5 percentage point increase in this indicator attributable to the intervention (Table 3). In the intention-to-treat analysis, the DID was significant, but much smaller at 27.7 percentage points (Table 4).
3. **Discussion of desired family size with one's spouse.** According to the results of the per-protocol analysis, the proportion of women that reportedly discussed desired family size with their spouse in the last 6 months was higher in the control group (74.6%) than in the intervention group (65.2%) at pre-study. At post-study, the indicator remained practically unchanged in the control group (66.7%) but increased in the intervention group (98.5%). The per-protocol DID estimate is 41.2 percentage points (Table 3), again indicating a significant positive effect of the intervention. Whereas the intention-to-treat estimate is much smaller (15.4 percentage points; Table 4), it remains nonetheless significant.
  The intervention appeared to increase the proportion of women who discussed desired family size and contraceptive methods with their spouse.
4. **Discussion of contraceptive methods with one's spouse.** The per-protocol analysis revealed that discussion of contraceptive methods with their spouse became more prevalent between pre-study and post-study in both the control (from 43.0% to 49.7%) and intervention (46.4% to 75.8%) groups. The DID estimate was marginally significant at 22.7 percentage points (Table 3). In contrast, the estimate from the intention-to-treat analysis was not significant (Table 4).
5. **Rejection of the misconception that contraceptive methods can harm the womb.** Results of the per-protocol analysis showed increased rejection of this misconception in the intervention group between pre-study (50.6%) and post-study (78.8%). In contrast, in the control group, proportionally fewer women (43.9%) at post-study rejected the misconception than at pre-study (64.1%). The per-protocol DID estimate stood large and significant at 48.4 percentage points (Table 3). The intention-to-treat estimate was smaller at 22.7 percentage points but still very significant (Table 4).
6. **Current use of modern contraceptive methods.** Whereas the use of modern contraceptive methods increased conspicuously in the intervention groups (from 28.8% at pre-study to 63.6% at post-study), it remained at the same level in the control group (32.7%) at both time points. The estimated DID was 34.8 percentage points using the per-protocol approach (Table 3) and 14.8 percentage points using the intention-to-treat analysis (Table 4).

### Attrition

One major challenge encountered during the course of the study was the high attrition rate. A large number of women were recruited, but many of them did not engage at all with the platform and a significant number dropped out. Field workers recruited a total of 794 women (401 in the intervention group and 393 in the control group) into the study. This number included 641 originally recruited and 153 replacements. Of this number, only 559 (221 in intervention and 338 in control groups) took the Welcome Call and/or initiated the pre-intervention survey. The rate of noninitiation was higher among the women recruited into the intervention group (44.9%) than for their peers recruited into the control group (13.7%). The number of women that participated in the post-intervention survey was 92 for intervention and 158 for the control, a loss to follow-up (relative to study initiation) of 58.4% and 53.3%, respectively.

One major challenge encountered during the study was the high attrition rate.

The differences in the pre-study sociodemographic characteristics between the women that participated in the post-study and their peers that were lost to follow-up are presented in Table 5. Among the women who participated in the intervention, the 2 groups were not significantly different in terms of age, marital status, and parity. The average age was 26.6 years in the group that participated in the post-study survey and 26.2 years in the lost-to-follow-up group. Mean parity was 2.43 for the post-study group compared with 2.08 for the women lost to follow-up. In contrast, the 2 groups were significantly different in terms of religion and, to some extent, education. The differences by religion were such that 73.9% of the post-study group was Muslim compared with only about 60.1% of the lost to follow-up group. The difference by education was marginally significant: whereas 39.1% of the post-study group had tertiary education, only 28.1% of the women that were lost to follow-up did. In the control group, there were no significant sociodemographic differences between the women who participated in the post-study survey and their peers who were lost to follow-up.

**TABLE 5.** Pre-Study Sociodemographic Characteristics of Study Participants, by Whether They Participated in the Post-Study Survey, Kaduna, Nigeria, 2017

Sociodemographic Indicator	Participated in Post-Study Survey (n=92)	Lost to Follow-Up: Did Not Participate in Post-Study Survey (n=129)	*Z* (or *t*)/*P* for Difference Between Groups
**Intervention group**			
Mean age, years	26.6	26.2	0.653/.51
Currently married, %	55.4	52.3	0.453/.56
Tertiary education, %	39.1	28.1	1.716/.09
Muslim, %	73.9	60.1	2.123/.03
Mean parity	2.43	2.08	1.145/.25
**Control group**			
Mean age, years	27.2	26.8	0.777/.44
Currently married, %	57.3	57.0	0.063/.95
Tertiary education, %	35.4	27.9	1.470/.14
Muslim, %	59.7	54.1	1.052/.29
Mean parity	2.44	2.43	0.011/.99

## DISCUSSION

Using longitudinal data, the analyses presented in this manuscript show that an IVR-based approach using drama is a viable option for promoting positive ideation related to family planning and increasing contraceptive use in Nigeria. The intention-to-treat DID results indicate that the intervention led to significant increases in contraceptive use and related ideational variables. This finding is consistent with prior reports on the use of mobile technologies in other settings. For example, in a study in Johannesburg, South Africa, Coleman and his colleagues^38^ assessed the effects of an SMS-based digital health intervention among pregnant women with HIV and found that the women that received the intervention had higher odds of obtaining the recommended amount of antenatal care visits, higher odds of normal vaginal delivery, and a lower odds of having a baby with low birth weight. Similarly, an SMS-based intervention in Nairobi, Kenya, was found to have led to increased exclusive breastfeeding and postpregnancy contraceptive use.^52^ In Zanzibar, Lund and her colleagues^45^ found that a mobile phone intervention increased skilled delivery attendance in urban but not in rural areas.

An IVR-based approach using drama is a viable option for promoting positive ideation related to family planning and increasing contraceptive use.

Results from the evaluation of the *Smart Client* digital tool reveal some of the challenges of using digital health to disseminate health information. Some of these lessons have been highlighted in previous studies,^40^^,^^47^^,^^53^ while others are specific to the *Smart Client* digital tool. Such challenges might have contributed to the high attrition rate in the study. First, a high rate of noninitiation or nonengagement with the platform was present among the women who were recruited into the study. The noninitiation rate was considerably higher among the women recruited into the intervention group compared with those recruited into the control group. One possible explanation for the higher noninitiation rate for the intervention group is the intensity of the intervention. At recruitment, the intervention group was made aware there would be 17 calls, whereas the control group was informed that they would receive only 2 calls. A second challenge was that among the women in the intervention group who engaged with the platform, a large proportion did not complete the intervention.

Several other factors might have contributed to the high attrition rate, especially the noninitiation problem. For multiple reasons, a delay occurred between recruitment and when the calls began. Another issue, as reported by participants during follow-ups, was that the beginning of the calls sounded like automated calls from a marketer. For this reason, some participants did not complete the calls. Furthermore, some participants cited technical issues with the platform as being demotivating. These issues included calls not being sent out at the correct time due to network problems and SMS messages not being delivered to all participants who used a particular mobile network. The high attrition rate due to technical issues is not unique to the *Smart Client* tool, and studies have identified this problem in other digital health interventions. For example, a study in Ghana^40^ found that due to problems related to technical functionality, only about a quarter of the health messages sent by the Mobile Technology for Community Health's mobile midwife program were received by the intended audience. Similarly, in Malawi, between 54% and 64% of SMS messages and between 27% and 38% of voice messages were successfully delivered to the intended audience.^42^ In contrast, registered users in the MomConnect mHealth program in South Africa received over 80% of the messages sent by the program.^40^ The developers of IVR platforms have also recognized the issue of attrition and noninitiation, especially in automated surveys, and for this reason, overrecruitment is commonly recommended.

With a study design that relied on the IVR platform to collect endline data, the high attrition rate could have significantly hampered the ability to make inferences. Fortunately, the study design used a prevalence of spousal communication (50%) that provided maximum variability to calculate the required sample size. The prevalence of spousal communication about family size among the intervention group turned out to be much lower than 50%, and it increased by more than the anticipated 15 percentage points at post-study. With these parameters, the reduced sample size at post-study still afforded us a 97.7% power to make inferences about the effects of the intervention. Furthermore, given the observed parameters, a repeated sample of 51 respondents is sufficient for a power of 80%. Nevertheless, the potential for noninitiation and high drop-out rate is a problem that should be accounted for when using mobile phone technology in the context of health behavior change interventions.

The high attrition rate was a problem, but the study design enabled calculation of a sample size that allowed overcoming it.

Some steps could be taken to minimize attrition and increase participation. For example, adequate measures should be put in place to minimize the delay between participants' recruitment and the start of the intervention; ideally the delay should not be more than a few days. In addition, it is important that the calls do not start with a sound like an automated call from a marketer. It could be beneficial to inform participants at the time of recruitment what the opening segment of the calls will sound like. Participants should be asked or given assistance to program the platform phone number into their phone at the time of recruitment so they will recognize the incoming call. Further, to avoid potential attrition due to technical issues with the platform, intensive testing should be conducted prior to wide-scale use. In the pilot test reported in this manuscript, participants were asked to select a convenient window of time for them to receive the calls. For each call, the system was automated to call back participants up to 6 times within this window of time. These accommodations added a layer of complication to the system that resulted in some groups not receiving calls or SMS messages. To avoid this problem, call scheduling options should be standardized for all participants.

### Lessons Learned

This pilot test yielded some valuable lessons learned, including those related to tool development and implementation as well as those connected with the evaluation of effects.

The pilot test yielded valuable lessons learned, including those related to tool development and implementation and evaluation of effects.

#### Tool Development and Implementation

- During the pretesting of the tool, participants expressed interest in more content. However, the analysis of listening patterns during this study indicates that most participants did not listen to additional segments of the calls. This gap between expressed interest and actual listening patterns has important implications for the design of the calls. Program implementers of future adaptations of the tool could consider shortening the content, eliminating segments, or splitting the segments into separate calls, so the calls are not so long. The modifications to the content, however, should be determined based on feedback from potential audience members that is ideally obtained through testing conducted by mobile phone delivery of the content to simulate the actual conditions of use.
- Although mobile phone penetration in developing countries has increased exponentially during the last few years, challenges remain with a mobile phone-based intervention due to the everyday issues faced by many owners and users of mobile phones. In the follow-ups with study participants, some commonly reported issues included sharing a phone with someone else, an inability to keep the phone regularly charged due to a lack of electricity, phones being lost or damaged, and switching phone numbers. These issues present various impediments due to the configuration of the platform and/or the design of the intervention. While these issues persist, some level of attrition must be assumed.
- Some study participants reported that they stopped listening due to their husband's disapproval of their participation in the study. While this problem was not widespread, it does indicate the challenge of implementing a tool targeting women in locations where men make decisions for their wives; however, women in these locations are likely to be in greater need of the information included in this tool. Future interventions could potentially include husbands so they are able to listen to the content and discuss it with their wives.
- Unanticipated problems occurred with the IVR platform, especially with some features (e.g., flashing and SMS) not functioning correctly. When such issues took more time than expected to fix, they may have contributed to the attrition observed in this study. Testing of the platform was conducted prior to initiating the study; however, more intensive testing of all the features of the platform and with all mobile networks should be conducted prior to widespread rollout of an intervention.
- No simple answer exists for the question of the cost effectiveness of the IVR approach compared to nondigital interventions. The costs associated with developing and implementing a tool such as *Smart Client* are highly dependent on various factors and decisions, but there are generally two main types of costs to consider: the costs of producing the content and the costs associated with the IVR platform.
  - Related to the content production, the HC3 team decided to work with a production house to record the content. This approach ensured that the quality of the audio content would be very high, both in terms of its presentation (with professional actors) and its sound quality (which is an important consideration when delivering via mobile phone). However, the content does not necessarily need to be professionally produced, and costs could be saved in this area. Another cost consideration related to the content development is the need to have content in multiple languages. This option is easy to accommodate on an IVR platform, which can be useful in locations where there are many local languages spoken; however, each language translation will add to the production costs.
  - The costs associated with setting up and maintaining an IVR platform can be very minimal (depending on the provider); however, the costs of the airtime minutes can add up very quickly especially when a call is long, which is another reason to reduce the amount of content. For the *Smart Client* testing and user study, the platform was set up to have all airtime costs charged to the project so that the calls would be free to users. This arrangement is the most common with use of IVR in developing countries not only because of the income level of audiences but also to encourage use of a tool that the audience may not be very familiar with. It is possible to set up an IVR platform that requires the listeners to pay a small amount to access the content. Willingness to pay a nominal fee was a concept explored by program implementers during pretesting but was not explored further in this study. Lastly, costs of airtime minutes and SMS messages can be reduced through negotiations with mobile network operators; however, this can be a lengthy process and may necessitate guaranteeing that the tool will have a very large audience (i.e., customers with known identities). These factors point to an IVR tool similar to *Smart Client* being cost effective at a medium scale for a targeted audience and for a limited period of time; it should reach an audience that is large enough to justify the content production and airtime costs, but not so large that airtime costs over an unlimited amount of time are enormous.

#### Evaluation of the Effects of the Tool

- Recruitment of participants into the study required specialized skills and a level of assiduity exceeding that for most other types of surveys. The recruiters needed to understand that the study participants would have to commit to receiving multiple program calls and stay in the program for up to 3 months. Moreover, recruitment required testing potential participants' numeracy skills and Hausa linguistic skills. Failure on the part of recruiters to completely apply recruitment guidelines might have contributed to the initial failure of some participants to engage with the platform and the high level of dropout along the way.
- Merging data from the various program calls with data collected during recruitment was difficult at best. This problem was due to the fact that the participant's telephone number, which was intended to be used as the unique identifier, was not consistent across calls. Moreover, the format used for recording the telephone number was not consistent between the data collected at recruitment and the data collected through the program calls. Harmonizing the formatting required considerable data manipulation skills and was time consuming. As a result, matching some cases across program calls was not possible. While it is impossible to avoid the situation of people using different cell phones across calls, evaluation and program teams as well as platform technicians should work together to ensure that the same formatting style is used for essential data fields across multiple data sources. For example, including or excluding the country code in a telephone number makes a lot of difference for the ability to match data from various sources.
- In this study, due to the high attrition rate, the post-intervention survey came close to not having a sufficient sample to permit inferences being made. Evaluation of future adaptations of the tool using randomized control trial or cluster-randomized control trial should anticipate and adequately plan for a higher than usual attrition rate.

## CONCLUSION

The *Beta Life* version of the *Smart Client* digital health tool offers a potentially effective approach for promoting positive contraceptive attitudes and encouraging women to adopt a modern contraceptive method. The tool could contribute to increasing contraceptive prevalence and reducing unmet need for contraceptives in Nigeria. Future adaptations of the tool should address the limitations connected with the number and length of program calls, its requirement for considerable numeracy skills in low literate settings, and the issues related to recruitment and initiation. Additional information about the tool, including full scripts and an Adaptation Guide can be found at https://healthcommcapacity.org/technical-areas/family-planning/smart-client-smart-couples/.

## Notes

**Peer Reviewed**

**Funding:** This research was made possible through the generous support and funding from the United States Agency for International Development (USAID) through the Health Communication Capacity Collaborative Project under Cooperative Agreement #AID-OAA-A-12-00058.

**Disclaimer:** The opinions expressed in this manuscript are the authors' and do not reflect the view of USAID or of Johns Hopkins University.
